# Chemical Composition, Nutritive Value, Volatile Profiles and Antioxidant Activity of Coconut (*Cocos nucifera* L.) Haustorium with Different Transverse Diameter

**DOI:** 10.3390/foods11070916

**Published:** 2022-03-23

**Authors:** Yufeng Zhang, Jintao Kan, Minmin Tang, Fei Song, Niu Li, Youlin Zhang

**Affiliations:** 1 College of Food Engineering and Nutritional Science, Shaanxi Normal University, Xi’an 710119, China; zhangyufeng062@163.com; 2 Hainan Engineering Center of Coconut Further Processing, Coconut Research Institute of Chinese Academy of Tropical Agricultural Sciences, Wenchang 571339, China; kanjint@163.com (J.K.); tanghn18@163.com (M.T.); songfeijj@163.com (F.S.); liniu2008.happy@163.com (N.L.)

**Keywords:** haustorium, volatile profiles, antioxidant activity

## Abstract

In order to promote the development and utilization of coconut haustorium (CH). The basic chemical composition, volatile profiles and antioxidant activities of three haustoria with different transverse diameters were investigated. Results showed large coconut haustorium (LCH) contained more soluble sugar (47.10%) and reducing sugar (17.68%), while small coconut haustorium (SCH) possessed more ash (10.17%), protein (9.22%) and fat (5.03%). All CH were rich in potassium (4.06–4.69%) and phosphorus (0.39–0.50%). The fatty acid composition of SCH and amino acid composition of middle coconut haustorium (MCH) was more reasonable, which indicated its relatively higher nutritive value. Acids ranging from 26.90% to 60.82% were the dominant volatile components in CH, especially isobutyric acid whose relative content in SCH was up to 56.78%. The haustorium extract with polysaccharide as the main component has certain antioxidant activities, the half eliminating concentration (EC_50_ values) of LCH on hydroxyl radical and SCH on 2,2-diphenyl-1-picrylhydrazyl (DPPH) and 2,2′-azino-bis (3-ethylbenzthiazoline-6-sulphonic acid) (ABTS) radical were 8.33, 1.18 and 2.44 mg/mL, respectively. These results provided a reference for the development and utilization of different CH as a raw material in functional food or dietary additives.

## 1. Introduction

Hainan province is the main coconut producing area in China with a planting area of 34.40 thousand hectares and an annual yield of coconut fruit of 227 million [1]. Moreover, Hainan province also has a complete processing industry chain with an annual processing capacity of more than 2.5 billion coconuts and an output value of more than 20 billion yuan. This means that Hainan province has to import a large number of coconuts from Indonesia, Vietnam and other Southeast Asian countries every year. However, due to the impact of labor shortage and the decline of agricultural product prices, the coconut harvest cycle in Southeast Asia has been extended to 90–120 days in recent years, resulting in a coconut germination rate of 5–8% after long-distance transportation to China [2]. In the sprouting coconuts, there is a spongy organ named as haustorium which can mobilize nutrients from coconut water and meat to nourish the germinating embryo and fill the entire water cavity in 20–24 weeks after germination [1,2,3,4]. Therefore, Hainan may produce about 10 thousand tons of haustorium every year because of the main processing materials of meat and shell. 

Coconut haustorium was called “coconut treasure”, “coconut heart” and “coconut apple” because of its sweet and crisp taste in Hainan province. Moreover, most of the existing reports on haustorium focus on the changes of morphological structure and certain substances during coconut germination. For example, the surface tissues of haustorium will bear undulating structures attached to the gradating endosperm and accumulate a large amount of sucrose and starch to form starch grains and oil droplets [5]. Moreover, the cell wall surrounding the epithelial cells is very thick with constrictions, while the cytoplasm is less electron-dense without the fine structure of the endoplasmic reticulum [3]. The weight, medium and long chain fatty acid content of the haustorium increased gradually within 4 months of coconut germination, and C_16:0_, C_18:0_, C_18:1_ and C_18:2_ in triacylglycerol and fat in total lipids of haustorium increased significantly during the germination of 4–5.5 months [6,7]. Moreover, the activities of superoxide dismutase (SOD) and peroxidase (POD) in haustorium increased, while the contents of soluble protein and polyphenols decreased gradually during the process of the haustorium filling the entire cavity [1,8]. In addition, there were also a few reports about the nutritional components and antioxidant activities of haustorium among 2 months of germination, and the effects of extrusion and peeling or not on the physicochemical properties of haustorium [2,4,9]. However, there are few systematic reports on what size of haustorium has higher functional activity and nutritional value including reasonable amino acid and fatty acid composition, rich minerals and so on, and whether there are differences in volatile components among the different haustorium, which is very unfavorable to the deep processing and utilization of haustorium.

Therefore, the aim of this paper was to systematically evaluate and analyze the basic components, nutritive value, volatile profiles and antioxidant activities of haustorium with different transverse diameters, so as to provide a basis for the development and utilization of coconut haustorium, and then promote the development of the coconut industry in Hainan Province.

## 2. Materials and Methods

### 2.1. Materials and Chemicals

Fresh haustorium was provided by the local coconut processing factory. All samples were obtained from the same batch of high variety coconuts from Kundian City, Indonesia. The single and mixed standards of fatty acid methyl ester and amino acid, ABTS and DPPH were purchased from Sigma-Aldrich Chemical Co., Ltd. (Shanghai, China). Germanium (Ge), Indium(In), Bismuth (Bi) and other multi-element mixed internal standards, calibration standard solution (1 g/L mixed standards of iron, sodium, potassium, calcium and magnesium, 10 μg/mL mixed standards of silver, aluminum, copper, molybdenum, zinc solution and other fifteen elements), mercury standard solution, other multi-element standard solutions, and nitric acid were purchased from Agilent Technologies, Inc. (Beijing, China). Other reagents and solvents, such as phenol, ethanol, chloroform, sulfuric acid, salicylic acid, ascorbic acid (Vc) and so on were obtained from Lianshi Yunshang Network Technology (Hainan) Co., Ltd. (Haikou, China).

### 2.2. Preparation of Coconut Haustorium Samples

After washing with distilled water to remove impurities, the coconut haustoria were divided into three types of small (<4.00 cm), medium (4.01–7.00 cm) and large size (>7.00 cm) according to the transverse diameter. The corresponding seeding length of germinating coconuts ranged from 0 cm to 5 cm, from 5 cm to 15 cm and more than 15 cm, respectively [1]. Then they were cut into uniform thin strips and dried at 60 °C to constant weight. Finally, the dried samples were crushed through a 100 mesh sieve, sealed and stored in a desiccator for further analysis. The obtained samples were named SCH, MCH and LCH, respectively.

### 2.3. Determination of Chemical Composition

Chemical compositions including moisture (AOAC 935.29), ash (AOAC 938.08), protein (AOAC 2001.11) and fat (AOAC 920.39) were determined according to the methods recommended by AOAC [10]. The soluble and reducing sugar was determined by the 3,5-Dinitrosalicylic acid assay and was described according to the previous report with glucose as a standard [11]. The glucose standard curve was y = 5.4502x − 0.0338, and the correlation coefficient (R^2^) was 0.9977.

### 2.4. Analysis of Nutritive Value

#### 2.4.1. Determination of Fatty Acids Composition

Haustorium oil extraction: 20 g samples and 400 mL petroleum ether was reflux extracted at 45 °C in a water bath for 24 h. The residue was collected and extracted twice again. The extracts were combined and evaporated at 40 °C under vacuum conditions to obtain haustorium oil.

Methyl ester preparation: 60 mg of haustorium oil was dissolved in 4 mL isooctane. Moreover, 200 μL of 2.0 M methanol containing 2% potassium hydroxide was added and shaken vigorously for 30 s under sealed conditions, and left to be clarified. Next, 1 g sodium bisulfate was added and shaken violently to neutralize the residual potassium hydroxide. The upper solution was transferred to the sample bottle for further chromatographic analysis.

Determination and analysis: Fatty acid composition of samples and standards were measured by an Agilent 7890A gas chromatograph (Wilmington, NC, USA) equipped with a Supelco SP-2560 capillary column (100 m × 0.25 mm, 0.20 μm) and hydrogen flame ionization detector (FID). The injector and detector temperatures were 270 °C and 280 °C, respectively. The temperature increasing program was as follows: the initial temperature was 100 °C with 13 min holding, followed by an increase from 100 to 180 °C (10 °C/min) and 6 min holding, 180 to 200 °C (1 °C/min) and 20 min holding, 200 to 230 °C (4 °C/min) and 10.5 min holding. Other conditions include nitrogen as a carrier gas, a split ratio of 100:1 and an injection volume of 1.0 μL. The individual fatty acids methyl esters were identified by comparing with that of standard. The fatty acid content was quantified as the percentage of total fatty acids in the sample.

#### 2.4.2. Determination and Analysis of Amino Acids Composition

Moreover, 0.1 g of sample and 10 mL 6 M hydrochloric acid in a sealed hydrolysis tube was filled with nitrogen for protection and hydrolyzed at 110 °C for 22 h. Then the hydrolysate was cooled, filtered and constant volume to 25 mL. Moreover, 1.0 mL of the filtrate was evaporated twice until dry. Finally, the residue was dissolved in 1.0 mL sodium citrate buffer solution (pH 2.2) and filtered through a 0.22 μm membrane for further analysis. Animo acid composition of the sample and standards was measured by L-8900 automatic amino acid analyzer (Hitachi, Tokyo, Japan) with a cation exchange resin analytical column. The chromatographic conditions are listed as follows: separation column temperature of 57 °C, reaction column temperature of 135 °C, a flow rate of buffer solution of 0.4 mL/min, a ninhydrin flow rate of 0.35 mL/min, injection volume of 20 μL. The detection wavelength and analysis time were 570 nm and 440 nm, 20 min, respectively. The same volume of mixed amino acid standard solution was used as the external standard. The concentration of amino acids in the sample was calculated by peak area. The content of each essential amino acid in the protein was conversed according to the protein content of each sample, and the essential amino acid score (EAAS) was the ratio between the content of essential amino acid in the protein content of the sample (mg/g protein) and that of the corresponding essential amino acid in the FAO/WHO reference (mg/g protein).

#### 2.4.3. Determination of Mineral Elements

Next, 0.25 g of sample and 6.0 mL of nitric acid were mixed in the digestion tank to predigest for 1 h. Then 2 mL of hydrogen peroxide was added to digest thoroughly in the CEM Mars 5 microwave digestion instrument (Matthews, NC, USA) by the following digestion procedure: heating from 0 to 120 °C within 8 min and holding for 2 min; from 120 to 160 °C within 5 min and holding for 5 min; from 160 to 180 °C within 5 min and holding for 15 min. The digestion solution was cooled and fixed to 100 g for further testing. The mineral elements were determined by an Agilent 7700X Inductively coupled plasma mass spectrometry (ICP-MS) (Wilmington, DE, USA) equipped with quartz concentric atomizer, double channel atomizer and integrated torch tube (2.5 mm spray tube). The working condition of the mass spectrometer was a radio frequency power of 1600 W, carrier gas flow rate of 1.0 L/min, a peristaltic pump flow rate of 0.1 rps, atomization chamber temperature of 2 °C, oxide index of 0.45%, double charge index of 1.01%. The standard curve of each element was drawn with the diluted standard concentration as the abscissa (X) and the ratio of the element to signal response value as the ordinate (Y); 5% nitric acid blank solution was determined for 11 consecutive times to calculate the standard deviation of each element, and the concentration corresponding to three and 10 times the standard deviation were taken as the limit of detection (LOD) and quantification (LOQ), respectively.

### 2.5. Determination of Volatile Profiles

Gas chromatography combined with an ion mobility spectrometer system (GC-IMS, FlavourSpec, G.A.S., mbH, Dortmund, Germany) equipped with an automatic headspace sampler unit (CTC-PAL, CTC Analytics AG, Zwingen, Switzerland) was used for volatile profile analysis of three samples. Firstly, 2 g of powder samples were placed into 20 mL headspace glass vials. Next, the samples were capped and incubated for 20 min at 85 rps and 60 °C with splitless mode. Then 200 μL of headspace gas was automatically injected into the injector at 85 °C. The volatile compounds were transferred into an FS-SE-54-CB-1chromatographic column (15 m × 0.53 mm) by nitrogen (99.99%) at a programmed flow as follows: initially 2.0 mL/min holding for 2 min, then linear increase to 10 mL/min from 2 to 10 min and 100 mL/min from 10 to 20 min, respectively. The drift gas of nitrogen (99.99%) was set as 150 mL/min at 45 °C. Each sample was measured for three replicates. The n-ketones C_4_-C_9_ as external references were used for calculating the retention index (RI) which was applied for the identification of volatile compounds by comparing them with standards in the GC-IMS library. The relative content of each volatile substance was calculated by peak area normalization.

### 2.6. Fourier-Transform Infrared (FTIR) Spectroscopy

KBr powder and the dried samples were mixed evenly and pressed into a pellet and measured on FT-IR spectroscopy (Tensor 27, Buruker Co., Ettlingen, Germany) in the wavenumber range from 400 cm^−1^ to 4000 cm^−1^.

### 2.7. Assay for Antioxidant Activities In Vitro

#### 2.7.1. Samples Preparation

Moreover, 1.0 g of sample was extracted with 5 mL of 80% (*v*/*v*) ethanol by continuous shaking for 30 min in the dark at 60°C. After centrifugation at 5000 g for 15 min, the supernatant was collected. The residue was re-extracted twice and the supernatant was combined and vacuum evaporated at 50 °C to dryness. The dried extracts were resuspended in 25 mL of distilled water to obtain the 40 mg/mL of the extract solution and stored at −20 °C.

#### 2.7.2. Determination of Radical Scavenging Activities

The DPPH, hydroxyl and ABTS radical scavenging activity of the extract with suitable concentration were evaluated by the reported method [12,13], and the ascorbic acid (Vc) as a positive control. Moreover, the half eliminating concentration (EC_50_ values) of samples and controls were calculated by SPSS 25.0 software. The content of polysaccharides, protein and total phenols of the extraction solution was measured by the phenol-sulfuric acid method using glucose as the standard [12], Bradford method using bovine serum albumin (BSA) as the standard [14] and Folin–Ciocalteau method with Gallic acid as a standard [15], respectively.

### 2.8. Statistical Analysis

All data were expressed as mean ± standard deviation and analyzed by one-way analysis of variance (ANOVA) in SPSS 25.0 software. The Microsoft Office 2007 and Origin 2021 software was used for drawing and data calculation. Differences at *p* < 0.05 were taken as statistically significant.

## 3. Results and Discussions

### 3.1. Analysis of Yield Data and Chemical Composition

Generally speaking, it took about 50 days for Indonesia’s mature coconuts to be picked, stripped and shipped to China. However, the transportation cycle had now been extended to more than 90 days because of the influence of the worldwide spread of COVID-19 and local labor shortages [1]. Moreover, these imported coconuts were usually stored in the warehouse of the Hainan factory for a short time before shelling. Therefore, the high temperature and humidity environment in the cabin and warehouse would inevitably lead to a sharp increase in the number of germinating coconuts. A total of 193 intact haustoria with a total mass of 6869.50 g were collected from a small container after removing the 40 damaged haustoria. Both in terms of mass (72.30%) and quantity proportion (65.28%), MCH accounted for the largest (Table 1). The average transverse diameter, longitudinal diameter and weight of SCH, MCH and LCH were significantly increasing (*p* < 0.05). A similar result could be seen in the previous report [1]. However, they fluctuated greatly, especially for the average weight of LCH (79.75 ± 25.57 g) and the average longitudinal diameter of SCH (3.91 ± 0.87 cm). This was consistent with the previous report that the average weight of haustorium collected from 2 months germinated coconut changed greatly (80.2–131 g) [2].

In terms of chemical composition, the soluble sugar of SCH, MCH and LCH were increased significantly (*p* < 0.05) from 33.77 to 47.10 g/100 g dry basis (Table 1). The reducing sugar content had the same trend, but there was no significant difference between MCH and LCH (*p* > 0.05). This was slightly different from the previous research that the content of sucrose increased slowly and then decreased, while the fructose and glucose basically remained unchanged during the growth of haustorium [1,5]. Besides, the decreasing protein content indicated that the expansion of haustorium was a process of protein consumption (*p* < 0.05). This was consistent with the previous report [8]. Moreover, the high content of ash showed that the consumption of mineral elements was small during haustorium expansion. Besides, the fat content of SCH, MCH and LCH were 5.03, 4.27 and 4.03 g/100 g dry basis, respectively. Which was similar to that of unpeeled haustorium (5.59 g/100 g dry basis), but higher than that of the peeled sample (3.01 g/100 g dry basis) reported by Smita, et al. [4]. It further indicated that the yellow skin on the surface of coconut haustorium was rich in fat [2,4].

### 3.2. Evaluation of Nutritive Value

#### 3.2.1. Analysis of Fatty Acid Composition

The fatty acid composition of the three samples was different (Table 2). The dominant fatty acid of SCH, MCH and LCH was palmitic acid (23.47%), lauric acid (28.05%) and lauric acid (25.35%), respectively. Moreover, there were abundant unsaturated fatty acids (UFA) including oleic acid (13.66–15.99%) and linoleic acid (7.61–16.94) as well. This was different from previous reports that lauric acid decreased from 35.88% to 25.01% and linoleic acid increased from 3.00% to 9.86% gradually when coconut germinated from the 10th to 22nd week [6]. This might be caused by the difference in experimental coconut variety and local climatic conditions. Moreover, it also indirectly showed that the accumulation of lauric acid mainly occurred in the early stage of coconut germination, and the consumption of lauric acid happened in the later stage. Because some other researchers found that medium and short chain fatty acids (C_8:0_–C_12:0_) showed a trend of first increase and then decrease with haustorium growth [1,7]. Generally speaking, the total fat intake accounts for 30–35% of the total energy of healthy adults, and the best ratio of saturated fatty acids (SFA), monounsaturated fatty acids (MUFA) and polyunsaturated fatty acids (PUFA) was 1:1:1. So the nutritional balance of oil from SCH was the best, followed by LCH and MCH. Moreover, they were all better than the virgin coconut oil prepared from coconut meat with the SFA/MUFA/PUFA of 100.60:7.09:1 and walnut oil by cold pressing of 1:1.51:7.70 [16,17].

#### 3.2.2. Analysis of Amino Acid Composition

As the basic unit of protein, the kind and content of amino acids often determine the nutritional value of protein. As the results are shown in Table 3, seventeen amino acids were detected in three samples, among which tryptophan was absent in the essential amino acids, and glutamic acid was the main amino acid ranging from 110.80 to 145.92 mg/g protein. Similar results were also found in the amino acid composition of protein fractions obtained from coconut cake [18]. However, Manivannan, et al. pointed out that the highest content of amino acid in mature haustorium was aspartic acid [2]. This difference might be caused by the different germination times of haustorium. In addition, the content of aspartic acid and arginine was relatively high, and that of cysteine was the lowest. The percentages of aromatic amino acids including phenylalanine and tyrosine and sulfur-containing amino acids including cysteine and methionine in total amino acids of SCH, MCH and LCH were relatively lower for 7.22%, 7.22% and 7.49%, 9.29%, 8.02% and 8.41%, respectively, which indicated the antioxidant activities of haustorium or its proteins content might be low [18,19].

From the perspective of nutritional value, three haustoria were not superior protein resources due to the lack of tryptophan in essential amino acids. However, the EAA of three samples ranged from 304.39 to 342.71 mg/g protein which was close to that of the FAO/WHO reference (328 mg/g protein) [2]. In addition, MCH with essential amino acid/total amino acid (EAA/TAA) of 41.10% and essential amino acid/nonessential amino acids (EAA/NEAA) of 69.79% was the closest to the superior protein standard with EAA/TAA of 40% and EAA/NEAA of 60% stipulated by FAO/WHO [20,21]. The EAAS of leucine, lysine, phenylalanine and tyrosine in all samples and other three amino acids including histidine, methionine and cysteine in MCH were less than 1.00. In particular, the EAAS of lysine (0.77), lysine (0.76) and leucine (0.71) in SCH, MCH and LCH were the lowest, which meant that they were the first limiting amino acid of each sample except the missing tryptophan. Meanwhile, the isoleucine, methionine, threonine and valine in all samples were in excess condition (EAAS ≥ 1.00). Therefore, it was necessary to be used with milk, egg, wheat, cornflour and other resources rich in the above insufficient amino acids when developing balanced nutrition food with haustorium as raw basis material [2,21].

#### 3.2.3. Analysis of Mineral Elements

Mineral elements play an important role in maintaining human health. Excessive or insufficient intake will lead to malnutrition or body dysfunction and diseases [22]. As in the results shown in Table 4, the R^2^ of the standard curve of all elements ranged from 0.9936 to 1.0000, indicating that the linear relationship of the standard curve of each element was good. In addition, the LOD and LOQ of each element were in the range of 0.00002–0.0405 mg/kg and 0.0001–0.1350 mg/kg, respectively, which indicated the determination method had a high sensitivity. Moreover, fifteen mineral elements including nine recommended mineral elements in dietary nutrients reference intake (DRIs) for Chinese residents at different ages consisted of five macro-minerals and four micro-minerals were detected in three samples [23]. Potassium was the highest macro-mineral which account for about 5% of the dry weight. Similar materials with high potassium content were also found in the shoots of bamboo ranging from 4.19% to 6.66% of the dry weight [22,24]. That was to say coconut haustorium and bamboo shoots could be used as dietary supplements for people over 7 years old whose demand for potassium was as high as 1500–2000 mg/d. Moreover, this might explain why ash content in the chemical composition mentioned above was as high as 10%, too.

As for other macro-minerals, the content of phosphorus, sodium and magnesium in SCH, MCH and LCH had a significant difference (*p* < 0.05). Moreover, SCH had the highest phosphorus content of 4977.82 mg/kg, while LCH possessed the highest amount of sodium (2418.60 mg/kg) and magnesium (1706.43 mg/kg). Calcium would gradually accumulate with the growth of haustorium. Moreover, they were all higher than that of commonly consumed vegetables, such as okra, potato and carrot [25,26]. These indirectly indicated that more sodium, magnesium and calcium might be consumed in the subsequent development of coconut seedlings. Compared with the recommended nutrient intake (RNI) value, the consumption of 100 g of each coconut haustorium could meet the needs of 0–1 year old infants for four micro-minerals including iron, zinc, manganese and copper elements.

Besides, six other mineral elements, such as boron and nickel were also detected in the three samples, especially the content of boron was basically constant between 10.86 and 11.99 mg/kg. Previous studies had pointed out that boron could affect the composition and function of blood, brain, kidney and bone systems by regulating the metabolism of calcium, copper, triglyceride and reactive oxygen species. Moreover, reasonable supplementation of boron had a positive significance in improving the treatment of postmenopausal women and elderly osteoporosis [27,28]. So coconut haustorium might be a potential boron supplement resource because the recommended consumption of boron by WHO was 1–13 mg/d, but its excessive toxicity should be avoided [29].

### 3.3. Analysis of Volatile Profiles

**Figure 1:**
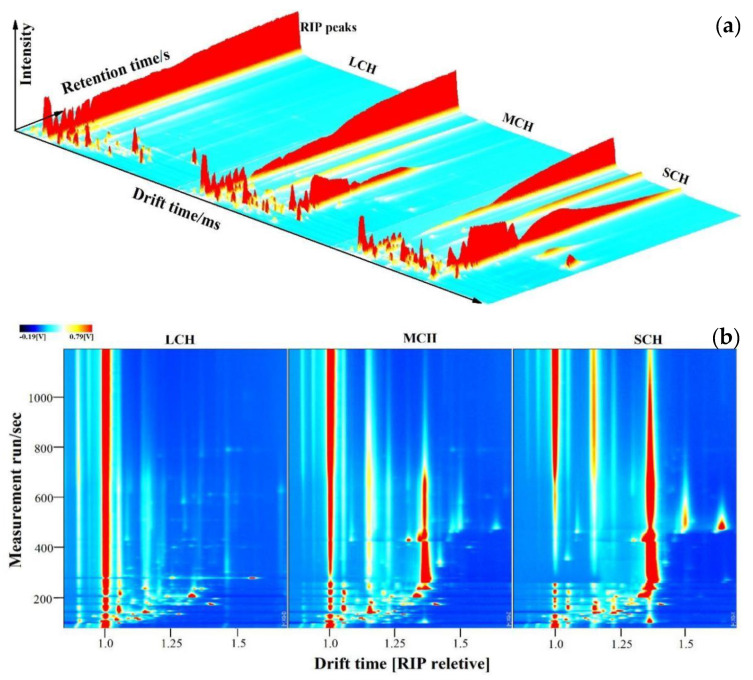
3D-chromatograms (**a**) and two dimensional (**b**) GC-IMS spectra of volatile profiles in coconut haustorium. (The background of two-dimensional (**b**) was blue, and the red vertical line at abscissa 1.0 was RIP peaks (reaction ion peak, normalized). The ordinate represents the retention time (s) of GC, and the abscissa represents the ion drift time (normalization). Each point on both sides of RIP represents a volatile compound.).

GC-IMS was a new flavor detection technology in recent years. It had the advantages of the high separation ability of gas chromatography (GC) and high resolution and sensitivity of ion mobility mass spectrometry (IMS). Moreover, it also had the characteristics of simple operation, fast analysis, visualization of flavor substances and so on, which made it widely used in food flavor analysis, quality detection and control [30,31,32]. However, the research on the volatile components of coconut haustorium had not been reported so far. Results showed that the volatile compounds in the three samples could be separated well in GC-IMS. The 3D spectra of volatile compounds were basically similar, but there were still great differences in local areas (the greater the difference of signal peak response at the same position, the greater the difference of the content of the substance in Figure 1a). This difference could be shown more intuitively by comparing the color difference at the same position in Figure 1b. The redder the color, the stronger the signal intensity of the substance, that was, the higher the relative content.

**Figure 2:**
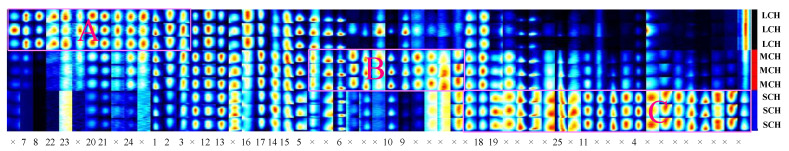
GC-IMS fingerprints of volatile profiles in coconut haustorium. (Each line represented all signal peaks selected from a sample. Each column represented the signal peak of the same volatile substance in different samples. The red boxes with letters A, B and C represented the characteristic substances of LCH, MCH and SCH, respectively. × represented the unidentified substance in the database. The number below the abscissa corresponded to the number of each volatile substance in Table 5).

In order to identify the characteristic flavor compounds more intuitively, 57 signal peaks shown in the fingerprint (Figure 2) were compared with the existing information of substances in the GC-IMS database. A total of 25 compounds were identified, which accounted for 71.22%, 75.76% and 70.79% of the total volatile profiles in SCH, MCH and LCH, respectively (Table 5). Most of these substances were small molecules that could be volatilized at room temperature [30]. Their retention times were between 110.73 s and 789.98 s, drift time ranged from 1.05 ms to 1.56 ms and the carbon chain length was between C_2_ and C_9_. Region “A” “B” and “C” which contained 14, 11 and 22 substances in Figure 2 represented the characteristic volatiles of LCH, MCH and SCH, respectively. There were 10 identified components in LCH samples, including ethanol (monomer and dimer), acetone, hexanal (monomer and dimer), pentanal (monomer and dimer), methylpyrazine, hexanoic acid and n-nonanal. While only three and five identified compounds existed in the characteristic volatiles of MCH and SCH, respectively.

In terms of the categories and relative contents, the identified 25 substances included eleven aldehydes, five acids, three alcohols, three ketones, two esters and one pyrazine. Aldehydes were the most abundant, accounting for 44% of the identified substances. However, acids had the highest relative content ranging from 26.90% to 60.82% (Table 5). This might be due to the accumulation of acid intermediate metabolites, such as propionic acid, hexanoic acid, isobutyric acid and acetic acid from the degradation of fat and protein in haustorium during the stage of haustorium expansion before seedling growth [1,6,8]. While the second largest group of volatiles were ketones for SCH (5.59%) and MCH (7.04%), aldehydes for LCH (17.43%). Specifically, isobutyric acid was the dominant volatile of SCH (56.78%) and MCH (50.68%), while that of LCH was acetoin (13.10%). Moreover, the relative content of seven compounds in three samples consisted of acetone, acetic acid, butyrolactone monomer, 2-methyl-propanal dimer, 2-methylbutanal monomer, 3-methylbutanal dimer and propionic acid dimer having a significant difference (*p* < 0.05).

**Figure 3:**
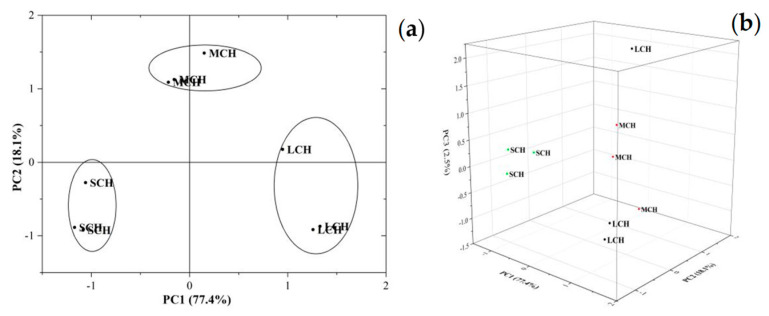
Two dimensional-PCA (**a**) and 3D-PCA (**b**) of volatile profiles in coconut haustorium.

The principal component analysis (PCA) of all identified substances showed that the cumulative variance contribution rates of the first three principal components were 77.41%, 18.14% and 2.53%, respectively (Figure 3b). In other words, the first and second principal components basically contained most of the information of the volatile components in three samples, they could be used for the comparative analysis of volatile components between different samples. Moreover, the close relative distance between the same group of samples, especially SCH and MCH, indicated that the detection repetition of samples was good. The obvious spacing between different groups showed obvious differences in volatile substances among them (Figure 3a). This was consistent with the previous results in Figure 3 and Table 5. These all showed that GC-IMS could distinguish LCH, MCH and SCH well by the difference in volatile substances.

### 3.4. Analysis of FTIR

**Figure 4:**
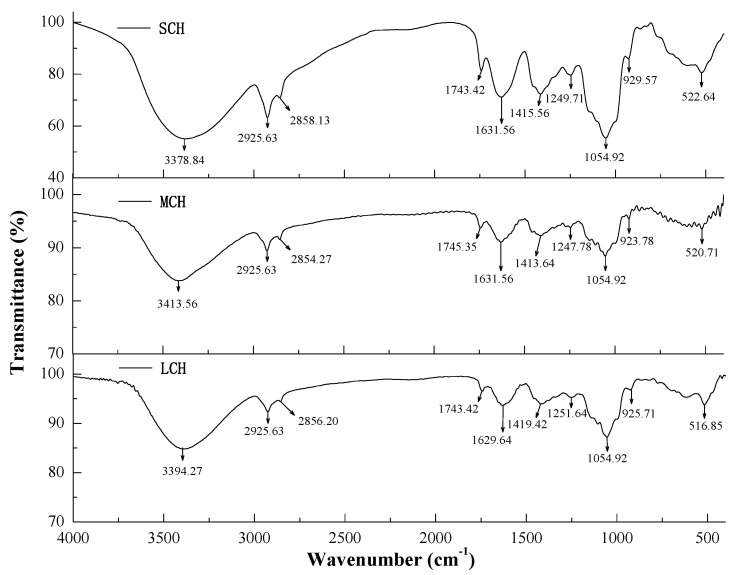
FTIR spectra of coconut haustorium.

The FTIR spectra of three samples were shown in Figure 4, strong absorption peaks at 3413.56–3394.27 cm^−1^ usually represented the stretching vibration of O-H [33,34]. Weak absorption peaks near 2925 cm^−1^ and 2850 cm^−1^ were related to the stretching vibration of C-H [4,35]. Peaks near 1740 cm^−1^ and 1630 cm^−1^ were assigned to the stretching vibration of ester and non-ester carbonyl C=O, respectively, which usually indicated the existence of protein and uronic acid [33]. The strong transmittance around 1415 cm^−1^ and 1250 cm^−1^ showed the variable angle vibration of C-H and stretching vibration of C-O, respectively [33,35]. Furthermore, the sharp peak at 1054.92 cm^−1^, weak absorption near 920 cm^−1^ and 520 cm^−1^ of fingerprint area were attributed to the stretching vibration of the β-pyranose ring [4,34,36]. On the whole, the FTIR spectra of CH were basically the same except for the low transmittance of SCH. Similar results could be seen in the peeled and unpeeled haustorium as well [4]. This indicated that the organic functional groups of SCH, MCH and LCH were similar and it was difficult for us to judge which kind of haustorium was used in its processing product by the FTIR spectrum.

### 3.5. Analysis of Antioxidant Activities In Vitro

Antioxidant activities in vitro including DPPH, hydroxyl and ABTS radical scavenging ability and the main active substances of the extracted solution were shown in Table 6. The EC_50_ value of samples on DPPH, hydroxyl and ABTS radical ranged from 1.18 to 1.94 mg/mL, from 8.33 to 15.52 mg/mL, from 2.44 to 5.15 mg/mL, respectively, and they all had a significant difference (*p* < 0.05). In addition, the scavenging ability of coconut haustorium extract on hydroxyl radicals was the strongest compared to that of ascorbic acid (Vc) among the three models. Especially for LCH at 8.33 mg/mL was equal to that of Vc at 141.82 μg/mL. As we all know, hydroxyl radicals are one of the most active and toxic reactive oxygen species (ROS) which can attack almost all biological macromolecules and induce oxidative damage [13,34]. So coconut haustorium might be a potential antioxidant resource used in the research and development of functional foods or drugs.

The main active components in the extract of three samples were shown in Table 6. The content of polysaccharides in all samples was the highest, followed by phenols and protein. Moreover, the contents of these substances were significantly different among the three samples (*p* < 0.05). However, it was still difficult to determine which substances played a leading role in their antioxidant activities because of the proven contribution of polyphenols, polysaccharides, peptides, some amino acids as well mineral elements on antioxidant activity [13,15,16,37,38]. For example, glutamate and arginine could play an antioxidant role in plants and food systems by scavenging free radicals, improving the activity of antioxidant enzymes and inhibiting the production of ROS [39]. Hydrophobic amino acids (HAA) containing alanine, valine, methionine, isoleucine, leucine, phenylalanine and proline in peptides and protein could block the process of free radical chain reaction by combining with oxygen or reducing the release of hydrogen atoms in the system, so as to play an antioxidant effect [13,40]. Moreover, HAA of SCH (297.11 mg/g protein) and LCH (300.83 mg/g protein) was higher than that of MCH, (277.05 mg/g protein) (*p* < 0.05). Besides, the excessive presence of metal ions, such as Fe^2+^ and Cu^2+^ was conducive to the conversion of superoxide anion and hydrogen peroxide into hydroxyl radicals, resulting in the risk of oxidative stress [13,16]. Therefore, it was necessary to separate and purify the main components and further explore their antioxidant activities in order to promote the further processing and utilization of coconut haustorium.

## 4. Conclusions

Coconut haustorium, a by-product of the coconut processing industry, was rich in soluble sugar (33.77–47.10%), ash (8.22–10.17%) and protein (5.23–9.22%). However, transverse diameter significantly affected its chemical composition, volatile profiles and antioxidant activities. MCH with the highest content of potassium (46,892.57 mg/kg) and iron (31.84 mg/kg) and reasonable amino acid composition of EAA/TAA (41.10%) and EAA/NEAA (69.79%), accounted for the highest mass (72.30%) and quantity (65.28%) proportion in germination coconut for processing. While SCH had better fatty acid composition for its SFA/MUFA/PUFA of 3.98:1:1.11. Acids (26.90–60.82%), aldehydes (2.68–17.43%) and alcohols (5.59–16.18%) were the major volatile profiles in all coconut haustorium. All extracts from coconut haustorium had a high content of polysaccharides (14.81–21.17 mg/mL) and a certain free radical scavenging capacity. The EC_50_ values of LCH on hydroxyl radical and SCH on DPPH and ABTS radical were 8.33, 1.18 and 2.44 mg/mL, respectively. These indicated coconut haustorium could be used as mineral element enhancers, raw and auxiliary materials of food or medicine and antioxidant resources for deep processing and utilization, so as to improve its added value and promote industrial upgrading.

## Figures and Tables

**Table 1:** Yield data and chemical composition of coconut haustorium ^1^.

	Parameter	SCH	MCH	LCH
Yield data	Average transverse diameter (cm)	3.34 ± 0.49 ^c^	5.33 ± 0.75 ^b^	7.44 ± 0.39 ^a^
	Longitudinal diameter range (cm)	2.10–6.80	4.00–8.00	5.60–8.10
	Average longitudinal diameter (cm)	3.91 ± 0.87 ^c^	5.70 ± 0.83 ^b^	6.78 ± 0.5 ^a^
	Range of weight (g)	5.59–25.84	14.87–112.56	43.51–130.47
	Average weight (g)	12.29 ± 4.98 ^c^	39.42 ± 17.76 ^b^	79.75 ± 25.57 ^a^
	Quantity proportion (%)	26.42	65.28	8.29
	Mass proportion (%)	9.12	72.30	18.57
Chemical composition	Moisture (g/100 g wet basis)	85.21 ± 1.40 ^bc^	85.24 ± 0.85 ^ab^	87.54 ± 0.95 ^a^
	Soluble sugar (g/100 g dry basis)	33.77 ± 0.24 ^c^	42.87 ± 0.04 ^b^	47.10 ± 0.11 ^a^
	Reducing sugar (g/100 g dry basis)	12.62 ± 0.08 ^c^	17.44 ± 0.05 ^ab^	17.68 ± 0.82 ^a^
	Ash (g/100 g dry basis)	10.17 ± 1.56 ^a^	8.55 ± 0.55 ^a^	8.22 ± 1.52 ^a^
	Protein (g/100 g dry basis)	9.22 ± 0.06 ^a^	6.03 ± 0.04 ^b^	5.23 ± 0.04 ^c^
	Fat (g/100 g dry basis)	5.03 ± 0.12 ^a^	4.27 ± 0.12 ^b^	4.03 ± 0.23 ^b^

^1^ Values are the mean ± SD. Different letters in right superscript of the same line showed significant differences (*p* < 0.05).

**Table 2:** Fatty acid composition of coconut haustorium ^1^.

Fatty Acid	SCH (%)	MCH (%)	LCH (%)
Caprylic acid (C_8:0_)	1.86 ± 0.10 ^c^	3.73 ± 0.13 ^a^	3.01 ± 0.03 ^b^
Capric acid (C_10:0_)	1.84 ± 0.09 ^c^	3.31 ± 0.11 ^a^	2.89 ± 0.08 ^b^
Lauric acid (C_12:0_)	17.80 ± 0.73 ^c^	28.05 ± 0.55 ^a^	25.35 ± 0.46 ^b^
Myristic acid (C_14:0_)	13.78 ± 0.40 ^bc^	15.88 ± 0.43 ^a^	14.48 ± 0.08 ^b^
Palmitic acid (C_16:0_)	23.47 ± 0.05 ^a^	19.20 ± 0.58 ^bc^	19.20 ± 0.12 ^b^
Palmitoleic acid (C_16:1n7_)	0.22 ± 0.01 ^a^	0.19 ± 0.03 ^a^	0.24 ± 0.02 ^a^
Stearic acid (C_18:0_)	6.09 ± 0.18 ^bc^	7.07 ± 0.30 ^a^	6.62 ± 0.11 a^b^
Oleic acid (C_18:1n9c_)	15.99 ± 0.45 ^a^	13.66 ± 0.95 ^bc^	15.28 ± 0.20 ^ab^
Linoleic acid (C_18:2n6c_)	16.94 ± 0.63 ^a^	7.61 ± 1.05 ^c^	11.37 ± 0.11 ^b^
α- Linolenic acid (C_18:3n3_)	1.26 ± 0.06 ^a^	0.62 ± 0.08 ^c^	0.93 ± 0.01 ^b^
Arachidic acid (C_20:0_)	0.55 ± 0.03 ^a^	0.47 ± 0.03 ^b^	0.46 ± 0.01 ^bc^
Eicosenoic acid (C_20:1_)	0.2 ± 0.01 ^ab^	0.22 ± 0.01 ^a^	0.17 ± 0.01 ^c^
SFA	65.38 ± 1.15 ^c^	77.71 ± 2.13 ^a^	71.99 ± 0.35 ^b^
MUFA	16.41 ± 0.45 ^a^	14.06 ± 0.98 ^bc^	15.68 ± 0.17 ^ab^
PUFA	18.22 ± 0.69 ^a^	8.23 ± 1.14 ^c^	12.30 ± 0.12 ^b^

^1^ Values are the mean ± SD. SFA, MUFA and PUFA represented the abbreviation of saturated fatty acids, monounsaturated fatty acids and polyunsaturated fatty acids. Different letters in right superscript of the same line showed significant differences (*p* < 0.05).

**Table 3:** Amino acid composition and essential amino acid score of coconut haustorium ^1^.

Amino Acid	Content (mg/g Protein)			FAO/WHO Reference (mg/g Protein)	EAAS		
	SCH	MCH	LCH		SCH	MCH	LCH
Essential							
Histidine	18.91 ± 0.19 ^ab^	18.13 ± 0.49 ^bc^	25.01 ± 0.67 ^a^	19	1.00	0.95	1.32
Isoleucine	36.29 ± 0.30 ^a^	33.46 ± 0.04 ^bc^	33.51 ± 0.90 ^b^	28	1.30	1.20	1.20
Leucine	59.19 ± 1.38 ^a^	54.34 ± 0.15 ^ab^	46.80 ± 6.45 ^bc^	66	0.90	0.82	0.71
Lysine	44.51 ± 1.14 ^ab^	44.01 ± 0.05 ^bc^	46.59 ± 0.50 ^a^	58	0.77	0.76	0.80
Methionine	26.13 ± 1.51 ^a^	15.37 ± 0.86 ^c^	22.26 ± 1.61 ^ab^	25	1.25	0.75	1.33
Cysteine	5.06 ± 1.11 ^b^	3.34 ± 1.23 ^bc^	10.95 ± 1.39 ^a^				
Phenylalanine	34.93 ± 1.8 ^ab^	35.46 ± 0.22 ^a^	34.82 ± 2.91 ^abc^	63	0.87	0.85	0.97
Tyrosine	19.94 ± 0.85 ^ab^	18.00 ± 1.48 ^bc^	26.48 ± 3.22 ^a^				
Threonine	36.56 ± 1.95 ^abc^	39.66 ± 0.22 ^ab^	40.19 ± 0.75 ^a^	34	1.08	1.17	1.18
Valine	44.24 ± 0.45 ^b^	42.62 ± 0.1 ^c^	56.09 ± 0.01 ^a^	35	1.26	1.22	1.60
Nonessential							
Aspartic acid	66.09 ± 3.55 ^c^	84.57 ± 1.27 ^b^	102.15 ± 2.85 ^a^				
Serine	35.07 ± 1.61 ^c^	39.21 ± 0.18 ^ab^	40.95 ± 0.64 ^a^				
Glutamic acid	145.92 ± 4.51 ^a^	118.30 ± 1.06 ^ab^	110.80 ± 1.36 ^bc^				
Glycine	38.16 ± 0.68 ^ab^	36.65 ± 0.16 ^bc^	38.35 ± 0.52 ^a^				
Alaine	55.20 ± 0.12 ^b^	49.35 ± 0.09 ^c^	59.55 ± 1.45 ^a^				
Arginine	52.93 ± 0.02 ^c^	61.65 ± 2.94 ^b^	76.00 ± 0.46 ^a^				
Proline	41.13 ± 1.18 ^c^	46.45 ± 0.46 ^ab^	47.79 ± 1.89 ^a^				
EAA	325.75 ± 1.86 ^b^	304.39 ± 2.82 ^c^	342.71 ± 1.87 ^a^	328			
NEAA	434.50 ± 11.67 ^bc^	436.17 ± 5.79 ^b^	475.59 ± 4.46 ^a^				
TAA	760.25 ± 13.53 ^b^	740.57 ± 8.61 ^bc^	818.30 ± 2.60 ^a^				

^1^ Values are the mean ± SD. EAA, NEAA and TAA represented the amount of essential, nonessential and total amino acids, respectively. EAAS was the essential amino acid score. Different letters in right superscript of the same line showed significant differences (*p* < 0.05).

**Table 4:** Mineral elements of coconut haustorium ^1^.

Mineral Elements	Calibration Curve	R^2^	LOD (mg/kg)	LOQ (mg/kg)	SCH (mg/kg)	MCH (mg/kg)	LCH (mg/kg)	RNI (mg/d)
Macro-minerals								
Potassium	y = 1.001x + 33.31	0.9999	0.0405	0.1350	46,489.03 ± 385.94 ^ab^	46,892.57 ± 147.59 ^a^	40,555.94 ± 39.26 ^c^	350–2200
Phosphorus	y = 0.996x − 0.3331	0.9993	0.0214	0.0712	4977.82 ± 16.61 ^a^	4366.68 ± 51.20 ^b^	3908.77 ± 1.12 ^c^	100 *–720
Sodium	y = 0.9879x + 51.848	0.9999	0.0119	0.0395	1675.01 ± 8.90 ^c^	1956.56 ± 6.42 ^b^	2418.60 ± 20.59 ^a^	170–1600
Magnesium	y = 0.997x + 38.996	1.0000	0.0049	0.0162	1530.18 ± 11.51 ^c^	1651.65 ± 8.33 ^b^	1706.43 ± 6.93 ^a^	20 *–330
Calcium	y = 0.9851x + 55.76	0.9985	0.0485	0.1617	857.35 ± 8.61 ^c^	1130.25 ± 18.55 ^ab^	1155.65 ± 5.84 ^a^	200 *–1200
Micro-minerals								
Iron	y = 0.9909x + 47.555	0.9999	0.0039	0.0130	29.34 ± 1.35 ^b^	31.84 ± 24.68 ^a^	16.52 ± 0.66 ^c^	0.3 *–20
Zinc	y = 1.0059x − 0.7266	0.9989	0.0003	0.0009	29.40 ± 0.13 ^a^	21.14 ± 0.04 ^b^	16.43 ± 0.05 ^c^	2.0 *–12.5
Manganese	y = 1.0535x + 0.6334	0.9999	0.0002	0.0006	23.88 ± 0.13 ^c^	33.17 ± 0.10 ^b^	35.44 ± 0.20 ^a^	0.01 *–4.5 *
Copper	y = 0.9888x + 0.5056	0.9998	0.00002	0.0001	2.26 ± 0.007 ^c^	2.49 ± 0.05 ^b^	2.97 ± 0.03 ^a^	0.3 *–0.8
Other-minerals								
Boron	y = 1.0132x − 18.537	0.9951	0.0030	0.0100	10.86 ± 0.43 ^a^	11.99 ± 0.16 ^a^	11.40 ± 0.45 ^a^	
Rubidium	y = 0.9525x + 2.5483	0.9999	0.00002	0.0001	9.69 ± 0.003 ^c^	13.75 ± 0.16 ^a^	12.90 ± 0.02 ^b^	
Nickel	y = 1.0049x + 0.2771	0.9998	0.0028	0.0093	3.15 ± 0.04 ^a^	2.85 ± 0.04 ^bc^	3.06 ± 0.11 ^ab^	
Strontium	y = 0.952x + 4.4945	0.9999	0.00004	0.0001	2.25 ± 0.01 ^bc^	2.25 ± 0.02 ^b^	2.41 ± 0.01 ^a^	
Aluminum	y = 0.9248x + 1.4982	0.9936	0.0005	0.0016	1.92 ± 0.24 ^ab^	1.47 ± 0.01 ^bc^	2.23 ± 0.11 ^a^	
Tin	y = 0.9915x − 0.1772	0.9962	0.0002	0.0005	1.37 ± 0.02 ^bc^	1.44 ± 0.03 ^b^	2.79 ± 0.19 ^a^	

^1^ Values were the mean ± SD. LOD was limit of detection. LOQ was limit of quantification. RNI was the recommended nutrient intake, * was the adequate intake (AI). Different letters in right superscript of the same line showed significant differences (*p* < 0.05).

**Table 5:** Identified volatile compounds in coconut haustorium ^1^.

No.	Compound Name	CAS#	Retention Index	Retention Time (s)	Drift Time (ms)	Relative Amount (%)		
						SCH	MCH	LCH
1	Ethanol ^M^	64-17-5	525.5	112.023	1.04599	0.63 ± 0.04 ^bc^	1.59 ± 0.27 ^b^	4.75 ± 1.03 ^a^
2	Ethanol ^D^	64-17-5	526.4	112.467	1.13033	0.29 ± 0.02 ^bc^	0.70 ± 0.13 ^b^	2.40 ± 0.49 ^a^
3	Acetone	67-64-1	537	117.576	1.11781	0.39 ± 0.03 ^c^	0.92 ± 0.18 ^b^	2.48 ± 0.37 ^a^
4	2-Butanone	78-93-3	598	147.117	1.25226	1.39 ± 0.07 ^a^	0.73 ± 0.10 ^bc^	0.88 ± 0.09 ^b^
5	Acetoin	513-86-0	710.7	209.085	1.33076	3.78 ± 0.10 ^bc^	5.74 ± 0.70 ^b^	13.10 ± 1.78 ^a^
6	Acetic acid	64-19-7	583.3	140.009	1.15873	3.69 ± 0.17 ^c^	6.22 ± 0.63 ^b^	11.37 ± 0.85 ^a^
7	Hexanal ^M^	66-25-1	791.1	277.34	1.25828	0.03 ± 0.01 ^bc^	0.18 ± 0.09 ^b^	2.09 ± 0.79 ^a^
8	Hexanal ^D^	66-25-1	790.2	276.317	1.56437	0.02 ± 0.01 ^bc^	0.08 ± 0.05 ^b^	3.15 ± 1.46 ^a^
9	Butyrolactone ^M^	96-48-0	910.5	430.411	1.0828	0.09 ± 0.02 ^c^	0.75 ± 0.18 ^b^	1.44 ± 0.22 ^a^
10	Butyrolactone ^D^	96-48-0	908.7	427.132	1.30275	0.77 ± 0.06 ^bc^	1.94 ± 0.12 ^a^	0.89 ± 0.56 ^b^
11	2-Propanol	67-63-0	522.8	110.733	1.22213	0.36 ± 0.02 ^a^	0.34 ± 0.03 ^ab^	0.19 ± 0.06 ^a^
12	2-Methyl-propanal ^M^	78-84-2	568.3	132.746	1.10751	0.25 ± 0.02 ^bc^	0.58 ± 0.09 ^b^	1.64 ± 0.38 ^a^
13	2-Methyl-propanal ^D^	78-84-2	568.8	133.008	1.28396	0.55 ± 0.02 ^c^	0.90 ± 0.16 ^b^	1.62 ± 0.25 ^a^
14	2-Methylbutanal ^M^	96-17-3	665.2	179.655	1.15803	0.65 ± 0.04 ^c^	1.02 ± 0.08 ^b^	1.76 ± 0.23 ^a^
15	2-Methylbutanal ^D^	96-17-3	661.4	177.82	1.4016	0.31 ± 0.01 ^bc^	0.63 ± 0.13 ^b^	1.58 ± 0.43 ^a^
16	3-Methylbutanal ^M^	590-86-3	649.5	172.055	1.17311	0.10 ± 0.004 ^bc^	0.23 ± 0.03 ^b^	0.86 ± 0.24 ^a^
17	3-Methylbutanal ^D^	590-86-3	651.1	172.841	1.40764	0.61 ± 0.03 ^c^	1.31 ± 0.22 ^b^	2.65 ± 0.49 ^a^
18	Propionic acid ^M^	79-09-4	681.4	187.517	1.10751	0.16 ± 0.01 ^cb^	0.37 ± 0.05 ^b^	0.98 ± 0.19 ^a^
19	Propionic acid ^D^	79-09-4	682.5	188.041	1.26662	0.14 ± 0.02 ^c^	0.18 ± 0.02 ^b^	0.23 ± 0.01 ^a^
20	Pentanal ^M^	110-62-3	694.9	196.165	1.18292	0.05 ± 0.004 ^bc^	0.13 ± 0.03 ^b^	0.67 ± 0.21 ^a^
21	Pentanal ^D^	110-62-3	694.3	195.641	1.42347	0.06 ± 0.01 ^bc^	0.17 ± 0.05 ^b^	0.69 ± 0.16 ^a^
22	Methylpyrazine	109-08-0	823.7	315.365	1.0936	0.01 ± 0.005 ^bc^	0.03 ± 0.01 ^b^	0.31 ± 0.10 ^a^
23	Hexanoic acid	142-62-1	991	585.494	1.30102	0.05 ± 0.01 ^bc^	0.12 ± 0.03 ^b^	0.56 ± 0.16 ^a^
24	n-Nonanal	124-19-6	1105	789.975	1.47213	0.06 ± 0.01 ^bc^	0.22 ± 0.05 ^b^	0.71 ± 0.14 ^a^
25	Isobutyric acid	79-31-2	805.6	279.818	1.37849	56.78 ± 0.47 ^a^	50.68 ± 3.85 ^ab^	8.69 ± 0.95 ^c^
	Aldehydes (11)					2.68 ± 0.16 ^bc^	5.44 ± 0.97 ^b^	17.43 ± 4.76 ^a^
	Acids (5)					60.82 ± 0.29 ^a^	57.58 ± 3.1 ^ab^	26.90 ± 7.73 ^c^
	Alcohols (3)					1.29 ± 0.03 ^bc^	2.63 ± 0.38 ^b^	7.35 ± 1.45 ^a^
	Ketones (3)					5.59 ± 0.18 ^bc^	7.04 ± 0.44 ^b^	16.18 ± 2.14 ^a^
	Esters (2)					0.88 ± 0.06 ^c^	2.66 ± 0.16 ^a^	2.47 ± 0.70 ^ab^
	Pyrazines (1)					0.01 ± 0.01 ^bc^	0.03 ± 0.01 ^b^	0.31 ± 0.10 ^a^
	Total (25)					71.22 ± 0.25 ^b^	75.76 ± 0.0.69 ^a^	70.79 ± 0.75 ^c^

^1^ M and D represent the monomer and dimer, respectively. CAS# refer to the number assigned to each chemical substance by the Chemical Abstracts Service (CAS), an organization under the American Chemical Society. The numbers in brackets after aldehydes, esters, acids, ketones, esters and pyrazines indicated the quantity of substances. Different letters in the right superscript of the same line showed significant differences (*p* < 0.05).

**Table 6:** Antioxidant activities and main active substances of coconut haustorium extract ^1^.

Parameters		SCH	MCH	LCH	Vc *
EC_50_ value	DPPH	1.18 ± 0.01 ^c^	1.68 ± 0.02 ^b^	1.94 ± 0.01 ^a^	3.23 ± 0.03
	Hydroxyl	13.40 ± 0.08 ^b^	15.52 ± 0.26 ^a^	8.33 ± 0.02 ^c^	141.82 ± 1.32
	ABTS	2.44 ± 0.01 ^c^	3.27 ± 0.05 ^b^	5.15 ± 0.07 ^a^	12.03 ± 0.01
Active substance concentration	Polysaccharide	14.81 ± 0.13 ^c^	18.95 ± 0.13 ^b^	21.17 ± 0.20 ^a^	-
	Protein *	134.06 ± 1.38 ^a^	77.75 ± 1.09 ^b^	72.24 ± 1.33 ^c^	-
	Total phenols ^#^	150.84 ± 0.15 ^a^	127.86 ± 0.79 ^b^	89.92 ± 0.37 ^c^	-

^1^ The units of all results was mg/mL except for the data marked with * and ^#^ indicated the unit was μg/mL and μg GAE/mL, repectively. GAE was gallic acid equivalent. Different letters in right superscript of the same line showed significant differences (*p* < 0.05).

## Data Availability

All data presented in this study are available on request from the corresponding author. The data are not uploaded in publicly accessible databases.
